# DNVF Memorandum Partizipative Versorgungsforschung (Teil 1)

**DOI:** 10.1055/a-2665-0028

**Published:** 2025-10-27

**Authors:** Anna Levke Brütt, Sandra Borgmann, Eva Buchholz, Larissa Burggraf, Jennifer Engler, Florian Fischer, Tim Holetzek, Stefanie Houwaart, Andrea Icks, Franziska Jagoda, Sven Kernebeck, Christine Kersting, Theresia Krieger, Charlotte Kugler, Silke Kuske, Jonas Lander, Melanie Messer, Cathleen Muche-Borowski, Catharina Münte, Anna-Lena Röper, Sandra Salm, Daniel Schindel, Stefanie Schreiter, Sonja Teupen, Sebastian von Peter, Erik Farin-Glattacker

**Affiliations:** 1 Institut und Poliklinik für Medizinische Psychologie, Universitätsklinikum Hamburg-Eppendorf, Hamburg, Germany; 2 Department für Versorgungsforschung, Carl von Ossietzky Universität Oldenburg, Oldenburg, Germany; 3 Institut für Versorgungsforschung und Gesundheitsökonomie, Deutsches Diabetes-Zentrum, Leibnitz Zentrum für Diabetesforschung an der Heinrich-Heine-Universität Düsseldorf, Düsseldorf, Germany; 4 Institut für Versorgungsforschung und Gesundheitsökonomie, Centre for Health and Society, Medizinische Fakultät und Universitätsklinikum Düsseldorf, Heinrich Heine Universität Düsseldorf, Düsseldorf, Germany; 5 Deutsches Zentrum für Diabetesforschung e. V., Neuherberg, Germany; 6 Zentrum für Versorgungsforschung Brandenburg (ZVF-BB), Medizinische Hochschule Brandenburg Theodor Fontane, Brandenburg, Germany; 7 Abteilung Soziologie, Pädagogische Hochschule Schwäbisch Gmünd, Schwäbisch Gmünd, Germany; 8 Institut für Allgemeinmedizin, Goethe-Universität Frankfurt am Main, Frankfurt, Germany; 9 Sachgebiet Kommunikation, Wissenschaft und Gesundheitsförderung, Gesundheitsamt Frankfurt am Main, Frankfurt, Germany; 10 Bayerisches Zentrum Pflege Digital, Hochschule für angewandte Wissenschaften Kempten, Kempten, Germany; 11 Institut für Sozialmedizin und Epidemiologie, Medizinische Hochschule Brandenburg Theodor Fontane, Brandenburg an der Havel, Germany; 12 BRCA-Netzwerk e.V. – Hilfe bei familiären Krebserkrankungen, Bonn, Germany; 13 partieval – Vermittlung partizipativer Kompetenzen, Prozessbegleitung und Evaluation im Bereich Gesundheit GmbH, Aachen, Germany; 14 Institut für Epidemiologie, Sozialmedizin und Gesundheitssystemforschung, Medizinische Hochschule Hannover, Hanover, Germany; 15 Department für Pflegewissenschaft , Universität Witten/Herdecke, Witten, Germany; 16 Fachbereich Gesundheit, Fachhochschule Münster, Münster, Germany; 17 Institut für Allgemeinmedizin und Ambulante Gesundheitsversorgung (iamag), Universität Witten/Herdecke, Witten, Germany; 18 Fachbereich Medizinische Psychologie, Universität zu Köln, Köln, Germany; 19 Institut für Versorgungs- und Gesundheitssystemforschung (IVGF), Medizinische Hochschule Brandenburg Theodor Fontane, Brandenburg, Germany; 20 Forschungsbereich Versorgungs- und Implementierungsforschung, Fliedner Fachhochschule Düsseldorf, Düsseldorf, Germany; 21 Institut für Pflegewissenschaft, Universitätsklinikum Würzburg, Würzburg, Germany; 22 Lehrstuhl für Pflegewissenschaft, Universität Würzburg, Würzburg, Germany; 23 Institut für Allgemeinmedizin, Universitätsklinikum Hamburg Eppendorf, Hamburg, Germany; 24 Institut für Allgemeinmedizin und Palliativmedizin, Medizinische Hochschule Hannover, Hanover, Germany; 25 Deutsche Multiple Sklerose Gesellschaft, Bundesverband e.V., Hannover, Germany; 26 MS Forschungs- und Projektentwicklungs-gGmbH, Deutsches MS-Register, Hannover, Germany; 27 Institut für Medizinische Soziologie, Charité – Universitätsmedizin Berlin, Berlin, Germany; 28 Institut für Soziale Gesundheit , Katholische Hochschule für Sozialwesen Berlin, Berlin, Germany; 29 Klinik für Psychiatrie und Psychotherapie, Charité – Universitätsmedizin Berlin, corporate member of Freie Universität Berlin and Humboldt-Universität zu Berlin, Berlin, Germany; 30 Arbeitsgruppe Methoden in der Versorgungsforschung, Deutsches Zentrum für Neurodegenerative Erkrankungen e.V. (DZNE), Standort Witten, Witten, Germany; 31 Fakultät für Gesundheit, Department für Pflegewissenschaft, Universität Witten/Herdecke, Witten, Germany; 32 Universitätsklinik für Psychiatrie und Psychotherapie Rüdersdorf, Medizinische Hochschule Brandenburg, Rüdersdorf, Germany; 33 Sektion Versorgungsforschung und Rehabilitationsforschung, Universitätsklinikum Freiburg, Medizinische Fakultät, Universität Freiburg, Freiburg, Germany

**Keywords:** Partizipation, Versorgungsforschung, Patientenbeteiligung, Stakeholderbeteiligung, Bürgerbeteiligung, Participation, Health Services Research, Patient and Public Involvement, Stakeholder Involvement

## Abstract

Patient:innen als zentrale Akteur:innen der Gesundheitsversorgung sollen sich aktiv in Versorgungsforschungsprozesse einbringen können. Auch weitere Stakeholder – etwa Fachkräfte aus der Versorgungspraxis – sind für einen umfassenden partizipativen Ansatz von Bedeutung. In diesem DNVF Memorandum stehen partizipative Ansätze im Kontext der Versorgungsforschung im Mittelpunkt. Zunächst werden die Charakteristika partizipativer Versorgungsforschung beschrieben und ihr Entwicklungsstand sowie ihre Institutionalisierung in Deutschland dargestellt. Dabei werden auch das Potenzial und die Vorteile partizipativer Versorgungsforschung beleuchtet. Schließlich widmet sich das DNVF Memorandum zwei Querschnittsthemen, die für die Weiterentwicklung besonders relevant sind: der theoretisch-konzeptionellen Fundierung sowie der Erforschung von Effekten und Wirksamkeit partizipativer Ansätze.

## Abstract (en)

Patients, as central actors in healthcare, should be enabled to actively participate in health services research processes. In addition, other stakeholders, such as professionals from healthcare practice, are also essential for a comprehensive participatory approach. This DNVF memorandum focuses on participatory approaches in the context of health services research. It begins by outlining the key characteristics of participatory health services research and describing its current development and institutionalization in Germany. The DNVF memorandum also highlights the potential and benefits of participatory research. Finally, it addresses two cross-cutting topics that are particularly relevant for further development in this field: the theoretical and conceptual foundations, and the investigation of effects and effectiveness of participatory approaches.

## Präambel

Das vorliegende „Memorandum Partizipative Versorgungsforschung“ des Deutschen Netzwerks Versorgungsforschung e.V. (DNVF) gibt einen Überblick über den Entwicklungs- und Forschungsstand sowie die Umsetzung partizipativer Ansätze in der Gesundheitsversorgungsforschung 1 . Das Memorandum richtet sich an Versorgungsforschende und interessierte Patient:innen und Akteur:innen aus der Versorgungspraxis, an wissenschaftliche Einrichtungen, an wissenschaftliche Fachgesellschaften, an Forschungs- und Gesundheitsministerien, an Stiftungen und weitere Forschungsmittelgeber:innen.

Die Sprecher:innen der Arbeitsgruppe „Partizipative Versorgungsforschung“ des DNVF koordinierten den Schreibprozess, an dem Wissenschaftler:innen, Patient:innen(vertretende) und Akteur:innen aus der Versorgungspraxis aktiv beteiligt waren. Die Autor:innengruppe entwickelte das Konzept des Memorandums, woraufhin kleinere Arbeitsgruppen Entwürfe für die einzelnen Kapitel erarbeiteten. Diese wurden anschließend in einem mehrstufigen Prozess und unter Einbeziehung der Rückmeldungen von Mitgliedern des DNVF von der Autor:innengruppe überarbeitet und finalisiert.

Im Zentrum dieses Memorandums steht der Einbezug von Patient:innen 2 in die Versorgungsforschung, da diese im Mittelpunkt der Gesundheitsversorgung stehen. Darüber hinaus sind auch weitere Stakeholder – zum Beispiel professionelle Akteur:innen aus der Versorgungspraxis – relevant und sollten bei einem umfassenden partizipativen Ansatz berücksichtigt werden.

Im vorliegenden Teil 1 des Memorandums wird nach einer Einleitung (Kapitel 1), die die Charakteristika partizipativer Versorgungsforschung beschreibt, der Entwicklungsstand des Ansatzes dargestellt (Kapitel 2), wobei auch auf die Institutionalisierung in Deutschland eingegangen wird. Kapitel 3 befasst sich mit den Vorteilen und dem Potential partizipativer Versorgungsforschung und begründet die Relevanz des Ansatzes. In Kapitel 4 wird zur Skizzierung des Forschungsstands der partizipativen Versorgungsforschung auf zwei zentrale Querschnittsthemen eingegangen, die aus unserer Sicht für die weitere Verbreitung des partizipativen Ansatzes entscheidend sein werden: Die theoretische und konzeptionelle Fundierung von partizipativer Versorgungsforschung sowie Studien zu Effekten und der Wirksamkeit des Ansatzes.

Der separat veröffentlichte Teil 2 des Memorandums wird einen stärkeren Fokus auf die konkrete Umsetzung partizipativer Versorgungsforschung legen. Es wird auf Methoden und die Organisation partizipativer Studien sowie auf besondere Herausforderungen eingegangen. Diese Themen werden auch durch Präsentation von Forschungsbeispielen veranschaulicht.

## Teil 1

### Kapitel 1 – Einleitung – Was ist partizipative Versorgungsforschung?

Die Versorgungsforschung profitiert davon, die Expertise der Patient:innen und ihrer Angehörigen, Versorgenden sowie anderer Akteur:innen im Versorgungsprozess einzubeziehen. Sie können Interviewfragen beantworten oder Fragebögen ausfüllen, aber darüber hinaus auch im Sinne einer partizipativen Versorgungsforschung über den gesamten Projektverlauf oder in verschiedenen Projektphasen als Expert:innen an einem Forschungsprojekt mitwirken.

Um Beteiligung in der Versorgungsforschung zu spezifizieren, wurde von den Autor:innen des vorliegenden Memorandum in einem systematisierten Diskussionsprozess folgende Definition für den Begriff der partizipativen Versorgungsforschung erarbeitet:

„Partizipative Versorgungsforschung umfasst die wissenschaftliche Beschreibung, Analyse, Entwicklung, Evaluation und Veränderung gesundheitlicher Versorgung, bei denen die relevanten Akteur:innen, insbesondere Patient:innen und Versorgende, ihre Expertise aktiv in den Forschungsprozess einbringen und in möglichst vielen Forschungsphasen auf Entscheidungen Einfluss nehmen. Ziel ist die Patient:innenrelevanz der Studienergebnisse zu steigern und eine bedarfsgerechte, qualitativ hochwertige Gesundheitsversorgung unter Alltagsbedingungen zu ermöglichen.“

**Abb. 1:**
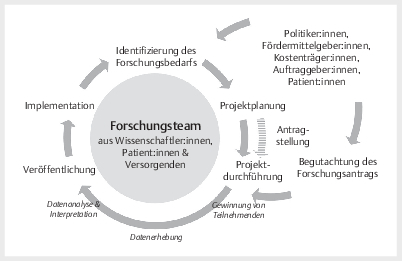
Typischer Ablauf eines partizipativen Forschungsprojekts in der Versorgungsforschung mit Forschungsteam, bestehend aus Ko-Forschenden und Wissenschaftler:innen.

Die partizipative Versorgungsforschung nutzt den Begriff der „Partizipation“, der ursprünglich zum Beispiel von Arnstein 1 beschrieben wurde. Mit Beteiligung ist in der partizipativen Versorgungsforschung nicht die Teilnahme als Proband:in oder Forschungsobjekt gemeint, sondern eine aktive Tätigkeit im Forschungsprozess. Dies beginnt bei der Mitwirkung an der Prioritätensetzung für Forschung. Die Zusammenarbeit innerhalb des und gemeinsam mit dem Forschungsteam erfolgt in möglichst vielen Phasen des gesamten Forschungsprozesses, von der Formulierung des Forschungsbedarfs und der Projektplanung sowie Antragstellung über die Projektdurchführung bis hin zur Veröffentlichung der Ergebnisse und der Implementation dieser in der Praxis (vgl. Abb. 1 ). Als Bezeichnung für zu beteiligende Patient:innen und andere Akteur:innen verwenden wir im Folgenden den Begriff „Ko-Forschende“. Oft wird auch der Begriff „Forschungspartner:in“ verwendet.

Gewinnbringend für die Versorgungsforschung ist vor allem die spezifische Expertise der Akteur:innen über Erkrankungen, die Versorgungsrealität und die Settings in denen versorgungswissenschaftliche Studien stattfinden. Patient:innen und Angehörige sowie Versorgende erleben die Gesundheitsversorgung mit allen Wirkungen und Nebenwirkungen, mit allen Systemkomponenten und ggf. Systembrüchen und sind daher Expert:innen in eigener Sache, deren Wissen die Forschung und Versorgung in vielerlei Hinsicht verbessern kann 2 3 . Dabei sind verschiedene Arten von Expertise zu differenzieren: individuelles Erfahrungswissen, kollektives Erfahrungswissen von bspw. Patient:innenvertretungen und Fachverbänden sowie Forschungserfahrung. Daraus ergibt sich ein breites Spektrum möglicher Ko-Forschender: Menschen mit individuellen Gesundheits- und Krankheitserfahrungen, ihre An- und Zugehörigen sowie andere Mitglieder des primären sozialen Netzwerks. Kollektives Wissen, das gesammeltem Wissen entspricht, kann über Mitglieder von Selbsthilfegruppen oder Vertretungen von Selbsthilfeorganisationen bzw. Organisationen i.S. § 140 f SGB V eingebracht werden. Forschungserfahrung durch eine wissenschaftliche Ausbildung kann das Wissen sinnvoll ergänzen. Eine ähnliche Gliederung ergibt sich für Versorgende, die individuelle Berufserfahrungen oder kollektive Erfahrungen aus Berufsverbänden und Fachgesellschaften einbringen können. Die verschiedenen Erfahrungen können in einer einzelnen Person gebündelt sein oder im Forschungsteam verteilt vorliegen.

In der partizipativen Versorgungsforschung sind verschiedene Beteiligungsintensitäten möglich. In Anlehnung an Leiter- und Stufenmodelle der Beteiligung 1 4 5 reichen diese von Konsultieren (z.B. Expert:innenbefragung) oder Involvieren (z.B. Projektbeirat) bis hin zu Kollaborieren, wo man intensiv voneinander lernt 6 . „Ignorieren“ und „Informieren“, die als Stufen in diesen Modellen beschrieben werden, sind keine Beteiligungsformate. Partizipation setzt einen aktiven Beitrag der Ko-Forschenden voraus. Ein besonderer Fall ist die selbstbestimmte/ betroffenenkontrollierte Forschung, die auch Formen der Zusammenarbeit einschließt, in der Akteur:innen der Selbstvertretung als Auftraggebende von Forschungsprojekten fungieren (z.B. 7 ).

Darüber hinaus sollte die Partizipation in der Versorgungsforschung sich nicht auf Konstellationen beschränken, in denen Forschungsteams unter Berücksichtigung verschiedener Expertisen zusammenarbeiten. Partizipation kann auch über das Zusammenwirken von Politiker:innen, Fördermittelgeber:innen, Kostenträger:innen, Auftraggeber:innen und Patient:innen zur Legitimierung von Förderausschreibungen, sowie zur Überprüfung der Mittelvergabe und der Sicherung der Implementierung von Versorgungsformen beitragen 8 .

### Kapitel 2 – Entwicklungsstand der partizipativen Versorgungsforschung

Die Bedeutung der Beteiligung der Gesellschaft an der Forschung ist mittlerweile in vielen Wissenschaftsbereichen erkannt. Die Fördermittelgeber:innen für Versorgungsforschung waren zunächst eher zurückhaltend, Bürger:innen oder Patient:innen aktiv an Projekten mitwirken zu lassen, inzwischen sind Elemente der partizipativen Forschung jedoch zunehmend Gegenstand allgemeiner Fördermaßnahmen 9 . Das Bundesministerium für Bildung und Forschung (BMBF) setzt Anreize, um partizipative Elemente (z.B. gemeinsames Lernen zwischen Forschenden und nicht wissenschaftlichen Partner:innen) in den Forschungsprozess zu integrieren. Auch in Projekten, die vom Innovationsausschuss beim Gemeinsamen Bundesausschusses (G-BA) aus Mitteln des Innovationsfonds Versorgungsforschung gefördert werden, sollen Patient:innen aktiv einbezogen werden. Herausfordernd ist diesbezüglich weiterhin die Struktur von Forschungsförderung, die oft nicht der ergebnisoffenen Arbeit partizipativer Forschungsprozesse entspricht. Das auf Initiative des Bundesministeriums für Bildung und Forschung initiierte Forum Gesundheitsforschung unterstützt den organisationsübergreifenden Dialog, um die Beteiligung von Patient:innen zu etablieren. Darüber hinaus haben Projektträger der Gesundheits- und Versorgungsforschung erste Publikationen bereitgestellt, die sich insbesondere an Wissenschaftler:innen richten und Handlungsempfehlungen zur aktiven Patient:innenbeteiligung enthalten 10 .

Auch wenn die Offenheit für partizipative Forschungsansätze in Deutschland in den letzten Jahren gestiegen ist, ist die partizipative Versorgungsforschung immer noch weniger institutionalisiert als im internationalen Raum. Es gibt zwar viele Patient:innenorganisationen und Selbsthilfeinitiativen mit struktureller Verankerung; diese sind jedoch bisher wenig in die Forschung einbezogen. International ist die partizipative Versorgungsforschung zumindest in Großbritannien, Kanada und den USA teilweise deutlich institutionalisierter, was z.B. daran erkennbar ist, dass offizielle Behörden und Institutionen Maßnahmen zur Förderung von Partizipation entwickeln und umsetzen. Dabei geht es nicht ausschließlich um partizipative Versorgungsforschung, sondern oftmals um übergreifende, allgemeine partizipative Strukturen und Prozesse, in die die Forschung einbezogen wird. Zudem zeigt sich, dass auch innerhalb einzelner Länder bzw. Regionen Institutionen jeweils eigene Ansätze, Frameworks, Guidelines etc. verfolgen bzw. veröffentlichen, bspw. die UK Standards for Public Involvement des National Institute for Health and Care Research (NIHR).

Die partizipative Forschung hat sich in Deutschland erst in den vergangenen 20 Jahren als Ansatz der Gesundheitswissenschaften entwickelt 5 . Sie ist zunehmend Gegenstand der Forschung in unterschiedlichen Sektoren und wird von zahlreichen Professionen durchgeführt (z.B. Public Health, Pflegewissenschaft, Medizin) 11 . Dies ist auch an der Grundlagenliteratur erkennbar, etwa durch die Fachbücher *Partizipative Forschung – Ein Forschungsansatz für Gesundheit und seine Methoden* 12 sowie *Partizipative Forschung: Einführung in die Forschungspraxis* 13 .

Unter den bislang etablierten Akteur:innen im deutschsprachigen Raum spielt das seit 2007 existierende Netzwerk Partizipative Gesundheitsforschung (PartNet) eine wesentliche Rolle. PartNet ging aus der internationalen Arbeitsgemeinschaft *International Collaboration for Participatory Health Research* (ICPHR) hervor 5 14 , fördert die Zusammenarbeit zwischen Wissenschaft und Praxis und entwickelt und veröffentlicht partizipative Methoden, etwa über die Beitragsreihe *PartNet Perspektiven. Beiträge zur partizipativen Forschung* .

In einigen Fachgesellschaften und Initiativen haben sich darüber hinaus Arbeitsgemeinschaften zur partizipativen Forschung gebildet, etwa innerhalb der Deutschen Gesellschaft für Sozialmedizin und Prävention (DGSMP). Bereits 2010 gründeten die Deutsche Vereinigung für Rehabilitation (DVfR) und die Deutsche Gesellschaft für Rehabilitationswissenschaften (DGRW) den Ausschuss „Reha-Forschung“, der in der Folgezeit mehrfach zur Teilhabe in der Forschung publizierte (z.B. 15 ). Das Aktionsbündnis Teilhabeforschung setzt sich für die grundlegende Weiterentwicklung der Lebenslagen von Menschen mit Behinderungen und chronischen Erkrankungen ein und fokussiert neben der Forschung zur Teilhabe auch die Teilhabe an der Forschung 16 . Das stärker grundlagenorientierte QUEST (Quality, Ethics, Open Science, Translation) Center for Responsible Research, angesiedelt am Berlin Institute of Health, befasst sich mit der Entwicklung und Unterstützung von Patient:innen- und Stakeholder-Beteiligung in der biomedizinischen Forschung.

Im Deutschen Netzwerk Versorgungsforschung bildete sich 2018 die Arbeitsgruppe Partizipative Versorgungsforschung, die sich u.a. mit Ansätzen der Partizipation in versorgungswissenschaftlichen Studien (z.B. durch Handlungsempfehlungen und Schulungen für Nachwuchsforscher:innen) befasst, aber auch mit der Entwicklung von Schulungen für Ko-Forschende sowie mit der Patient:innenbeteiligung auf Fachkongressen.

Darüber hinaus setzt sich der Deutsche Rheuma-Liga Bundesverband e.V. als Praxisinitiative für mehr partizipative Forschung ein und fördert seit 2015 mit Eigenmitteln partizipativ gestaltete Forschungsprojekte. Auf europäischer Ebene hat die europäische Rheuma-Liga (EULAR) ein Netzwerk von „Patient Research Partner“ (deutsch: "Forschungspartner") zur Umsetzung der partizipativen Forschung aufgebaut. Über dieses werden eine Broschüre und Referenzkarten zur partizipativen Forschung zur Verfügung gestellt, Auch die von Patient:innen-Vertreter:innen, Mediziner:innen und anderen Fachleuten gemeinsam betriebene Patienten-Experten-Akademie für Tumorerkrankungen (PEAK) bietet Aus- und Weiterbildungen an, um Patient:innen-Expert:innen auf ihre Aufgabe vorzubereiten und ihre individuelle Perspektive in Krebsforschung einzubringen.

### Kapitel 3 – Potential partizipativer Versorgungsforschung

Für die Notwendigkeit partizipativer Forschung wird aus unterschiedlichen Motivationen und Überzeugungen heraus argumentiert. So legitimieren sich Beteiligungsaktivitäten einerseits aufgrund normativer Werte: Partizipation als ethischer Grundsatz und moralische Verpflichtung, Partizipation zwecks Verwirklichung demokratischer Ideale wie Transparenz und Verantwortung oder Partizipation als Weg zum Empowerment von Bürger:innen und Patient:innen durch eine Mitverantwortung für Forschung und Leisten eines gesellschaftlichen Beitrag 17 18 19 . Andererseits kommen auch substantive Werte, z.B. inhaltlich-organisatorische Aspekte, zum Tragen. Zentral sind eine gesteigerte Qualität und Relevanz von Forschung durch eine stärkere Ausrichtung der Forschung an den tatsächlichen Bedarfen der Zielgruppe, eine verbesserte Umsetzbarkeit und Machbarkeit von Forschungsprojekten im adressierten Setting sowie eine niedrigschwellige und zielgruppengerechte Ergebnisverbreitung 17 18 . Ferner können Patient:innen aufgrund ihres Erfahrungswissens einen aktiven Beitrag zur Schaffung von Evidenz und Wissen sowohl innerhalb der Forschung als auch im gesamten Forschungsprozess leisten. Oft entstehen mit Hilfe partizipativer Forschungsansätze auch Forschungsfragen und Impulse, welche ohne eine Beteiligung von Betroffenen nicht zustande gekommen wären 15 .

Für Patient:innen bzw. Patient:innenvertretende, die oft Adressat:innengruppen eines “Forschungsproduktes” (z.B. einer digitalen Anwendung wie einer App) darstellen, kann partizipative Versorgungsforschung bedeuten, dass sie ein Mitbestimmungsrecht an jener Forschung erhalten, die sie direkt und indirekt in ihrer Lebenswelt betrifft 14 . Durch das Einbringen ihrer Erfahrungen in Forschungsprozesse und die Zusammenarbeit mit anderen Akteur:innen können sie Fähigkeiten, Ressourcen und Selbstbewusstsein im Sinne des Empowerments ausbauen 20 . Außerdem können sie sich zu Forschungs- und Gesundheitsthemen weiterbilden und ihren Zugang zu Informationen über den Umgang mit einer Erkrankung verbessern. Die Interessenvertretung ist eine wichtige Aufgabe der selbstorganisierten Patient:innenschaft, die sie durch Beteiligung ausüben kann.

Versorgungsforschende können durch die Einbindung der unterschiedlichen Perspektiven ein umfängliches Wissen über die Lebens- und Versorgungssituation der Adressat:innengruppen erlangen und zudem sicherstellen, dass ihre Forschung anwendungsbezogen und zielgerichtet ist 21 . Wenn sich professionelle Forschende auf partizipative Forschung einlassen, dann können sie oft auch eine Stärkung ihrer eigenen Kompetenzen und Fähigkeiten erfahren 22 23 . Darüber hinaus können bestehende Überzeugungen und Einstellungen, z.B. gegenüber der Adressat:innengruppe, hinterfragt und verändert werden 24 . Nicht zuletzt deshalb sollte partizipative Forschung auch Lehrinhalt verschiedener wissenschaftlicher Disziplinen sein, sodass Studierende schon frühzeitig mit der Idee der Partizipation in Berührung kommen und insbesondere das Reflektieren über die eigene Rolle im Forschungsprozess üben können.

Die Gesellschaft profitiert von Versorgungsforschung, die relevant und effizient ist. Durch die Orientierung an Patient:innen, sowohl hinsichtlich der Forschungsthemen als auch der Studiendesigns, trägt die partizipative Forschung dazu bei, unnütze Forschung zu vermeiden und Ressourcen sinnvoll einzusetzen 42 . Zudem können Versorgungsangebote direkt an den Bedarfen und Bedürfnissen derjenigen Personengruppen ausgerichtet werden, die diese in Anspruch nehmen 25 26 27 . Auf diese Weise können angemessene, qualitativ hochwertige, umfassende und möglicherweise auch kostengünstige Versorgungsformen entwickelt werden, die besser mit den Bedürfnissen der Betroffenen übereinstimmen 28 . Somit kann partizipative Forschung die Zusammenarbeit zwischen Forschung, Politik und patient:innenzentrierter Praxis verbessern 20 28 und zu einer nachhaltigen Nutzung von Forschungsergebnissen (z.B. neuen Versorgungsformen) führen 21 . Ebenso kann Beteiligung dazu beitragen, dass soziale, kulturelle und ökologische Ungleichheiten stärker berücksichtigt werden und die Versorgungsangebote den Aspekten der Nachhaltigkeit entsprechen 20 .

### Kapitel 4 – Aktuelle Querschnittsthemen der Partizipativen Versorgungsforschung

Der folgende Teil stellt zwei relevante Querschnittsthemen der partizipativen Versorgungsforschung dar: Arbeiten zur theoretischen und konzeptionellen Fundierung von partizipativer Versorgungsforschung sowie Studien zu Effekten und der Wirksamkeit des Ansatzes. Die langfristige Etablierung des partizipativen Ansatzes wird wesentlich davon abhängen, dass er auf der Basis einer handlungsleitenden theoretischen Konzeption der Partizipation erfolgt und dass sich der Nutzen für die Beteiligten sowie für Forschung und Versorgung belegen lassen.

Während im deutschsprachigen Raum bei vielen partizipativen Studien Fragen des Methodeneinsatzes und der praktischen Umsetzbarkeit im Mittelpunkt stehen, liegen international einige Arbeiten vor, die die **theoretische und konzeptionelle Fundierung** thematisieren. Im Rahmen eines Scoping Reviews wurde untersucht, welche Elemente den Modellen und Rahmenkonzepten der Partizipation von Patient:innen zugrunde liegen 29 . Im Kern zeigt sich, dass es vergleichsweise wenige konzeptuelle Überlappungen zwischen den herangezogenen Modellen gibt. Deshalb empfehlen die Autor:innen, dass Versorgungsforscher:innen bei der theoretischen Fundierung ihrer Arbeit den partizipativen Ansatz an den jeweiligen Studienkontext anpassen. Im Review werden u.a. Prinzipien zusammengetragen, die für partizipative Versorgungsforschung handlungsleitend sind 29 , etwa die frühe Einbeziehung im Forschungsprozess, Transparenz gegenüber den Forschungspartner:innen, und die Evaluation des partizipativen Vorgehens. Weitere Arbeiten zur konzeptionellen Fundierung partizipativer Versorgungsforschung liegen mit den Reviews vor 30 31 32 .

Ein Meta-Review konnte darüber hinaus 17 übergeordnete Prinzipien der Forschungspartnerschaft (z.B. „Die Partner:innen planen sorgfältig und reflektieren regelmäßig ihren strategischen Ansatz der Zusammenarbeit“) sowie 11 übergeordnete Strategien (z.B. „Biete praktische und emotionale Unterstützung für die zu Beteiligenden zur Überwindung von Hindernissen“) identifizieren 33 .

Viele internationale Arbeiten befassen sich mit Fragen der **Effekte und der Wirksamkeit partizipativer Versorgungsforschung** . Ein Sonderheft der Zeitschrift “BioMed Research International” beinhaltet mehrere Beiträge zum “Impact” von Partizipation und wie Wirkungen belegt werden können (vgl. Editorial von Wright, Salsberg and Hartung 34 sowie die deutschsprachig vorliegende Übersichtsarbeit von Allweiss, Cook and Wright 35 ). Weitere Reviews 36 37 berichten ähnliche Aspekte und lassen deshalb annehmen, dass die Wirkungen partizipativer Forschung über verschiedene Versorgungssettings bestehen. Allerdings weisen mehrere Autor:innen auf eine noch nicht hinreichende allgemeine Studienqualität und Evidenzlage hin, etwa weil die meisten partizipativen Primärarbeiten den eigenen Ansatz nicht evaluieren 38 . Grundsätzlich sollte eine Meta-Forschung über die Qualität, Wirkungen und Wirksamkeit von Partizipation dazu beitragen, die bisherigen und fortlaufenden Erfahrungen zu untersuchen, sodass zukünftige Forschungsvorhaben evidenzbasiert geplant und evaluiert werden können 39 .

Zum Thema Methodenvergleich zeigen Baumann und Brütt 40 in einem Vergleich eines partizipativen Workshops mit einem Delphi-Konsensusverfahren, dass diese Methoden ähnliche Ergebnisse erzielen, sich die Workshop-Teilnehmer:innen jedoch besser vorbereitet fühlen und auch andere Aspekte des Prozesses positiver bewerten als Teilnehmer:innen des Delphi-Verfahrens. Letzteres Verfahren erreichte die Zielgruppe effektiver, da die Befragung zeitlich flexibel ist und ohne zusätzlichen organisatorischen bzw. zeitlichen Aufwand für die Teilnehmenden auskommt.

Insgesamt besteht weiterer Bedarf an Forschung zur Wirksamkeit partizipativer Forschung, auch in Abhängigkeit von verschiedenen Partizipationsmethoden. Allerdings gibt es auch kritische Stimmen zur Fokussierung auf die Impact-Frage, die darauf hinweisen, dass ethische Beweggründe partizipativer Forschung – unabhängig von messbaren Effekten – zentral sind, um betroffene Personen zu stärken und eine gleichberechtigte Machtverteilung zu fördern 41 .

## Konsensusprozess

Das Memorandum „Partizipative Versorgungsforschung (Teil 1)“ wurde durch die AG Partizipative Versorgungsforschung im DNVF e.V. initiiert und unter Mitwirkung einer interdisziplinären Autor:innengruppe verfasst. Das abgestimmte Manuskript wurde entsprechend den Verfahrensvorgaben des DNVF an alle Mitglieder zur Kommentierung gegeben. Alle fristgerechten Kommentare wurden durch die Autor:innengruppe sorg fältig geprüft und entsprechend gewürdigt. Nach Abschluss des Konsensusverfahrens haben alle institutionellen Mitglieder die Möglichkeit gehabt, das Memorandum mitzuzeichnen. Die Freigabe des Dokuments erfolgte durch den Vorstand des DNVF. Das Memorandum „Partizipative Versorgungsforschung (Teil 1)“ wird von folgenden ordentlichen institutionellen Mitgliedern des Deutschen Netzwerks Versorgungsforschung e. V. getragen.

Mitglieder der Sektion „Fachgesellschaften“ (Sektion 1):

Deutsche Diabetes Gesellschaft e.V.

Deutsche Gesellschaft für Allgemeinmedizin und Familienmedizin e.V.

Deutsche Gesellschaft für Angiologie – Gesellschaft für Gefäßmedizin e.V.

Deutsche Gesellschaft für Arbeitsmedizin und Umweltmedizin e.V.

Deutsche Gesellschaft für Ergotherapiewissenschaft e.V.

Deutsche Gesellschaft für Gefäßchirurgie und Gefäßmedizin e.V.

Deutsche Gesellschaft für Gynäkologie und Geburtshilfe e.V.

Deutsche Gesellschaft für Hebammenwissenschaft e.V.

Deutsche Gesellschaft für Kardiologie – Herz- und Kreislaufforschung e.V.

Deutsche Gesellschaft für Kinder- und Jugendchirurgie e.V.

Deutsche Gesellschaft für Kinder- und Jugendpsychiatrie, Psychosomatik und Psychotherapie e.V.

Deutsche Gesellschaft für Medizinische Psychologie e.V.

Deutsche Gesellschaft für Mund-, Kiefer und Gesichtschirurgie e.V.

Deutsche Gesellschaft für Medizinische Soziologie e.V.

Deutsche Gesellschaft für Neurochirurgie e.V.

Deutsche Gesellschaft für Orthopädie und Unfallchirurgie e.V.

Deutsche Gesellschaft für Psychosomatische Medizin und Ärztliche Psychotherapie e.V.

Deutsche Gesellschaft für Rettungswissenschaften e.V.

Deutsche Gesellschaft für Rheumatologie und Klinische Immunologie e.V.

Deutsche Gesellschaft für Rehabilitationswissenschaften e.V.

Deutsche Gesellschaft für Senologie e.V.

Deutsche Schlaganfall-Gesellschaft e.V.

Deutsche Vereinigung für Sportwissenschaften e.V.

Gesellschaft für Phytotherapie (GPT) e.V.

Mitglieder der Sektion „Wissenschaftliche Institute und Forschungsverbünde“(Sektion 2):

Allgemeinmedizinisches Institut des Universitätsklinikums Erlangen

Abteilung für Allgemeinmedizin der Ruhr-Universität Bochum

BAG SELBSTHILFE e.V. Bundesarbeitsgemeinschaft

Bonner Netzwerk für Versorgungsforschung

Charité – Universitätsmedizin Berlin, Plattform – Charité Versorgungsforschung

Center for Health Care Research & Public Health

Institut für Allgemeinmedizin und Ambulante Gesundheitsversorgung (IAMAG)

Institut für Hausarztmedizin der Universität Bonn

Bayerisches Landesamt für Gesundheit und Lebensmittelsicherheit

LVR-Institut für Versorgungsforschung (LVR-IVF)

Sektion Versorgungsforschung und Rehabilitationsforschung, Universitätsklinikum Freiburg

Zentrum für Bevölkerungsmedizin und Versorgungsforschung

Zentrum für Evidenzbasierte Versorgungsforschung

Zentralinstitut für die kassenärztliche Versorgung in der Bundesrepublik Deutschland

Zentrum für Public Health und Versorgungsforschung

Mitglieder der Sektion „Juristische Personen und Personenvereinigungen“ (Sektion 3):

Deutsche Rheuma-Liga Bundesverband e.V.

Kassenärztliche Bundesvereinigung

## Hinweis

Dieser Artikel wurde gemäß des Erratums vom 18.02.2026 geändert. Erratum

Im oben genannten Artikel wurden mehrere Fehler in den Affiliationen 12, 13, 25 und 26 korrigiert. Die korrekten Affiliationen lauten: 12 BRCA-Netzwerk e.V. – Hilfe bei familiären Krebserkrankungen, Bonn, Germany 13 partieval – Vermittlung partizipativer Kompetenzen, Prozessbegleitung und Evaluation im Bereich Gesundheit GmbH, Aachen, Germany 25 Deutsche Multiple Sklerose Gesellschaft, Bundesverband e.V., Hannover, Germany 26 MS Forschungs- und Projektentwicklungs-gGmbH, Deutsches MS-Register, Hannover, Germany

Die Korrektur wurde in der Online-Version ausgeführt am 18.02.2026.

## Notes

Fazit Der vorliegende erste Teil des Memorandums führt in die Ziele, Prinzipien und Potentiale der partizipativen Gesundheitsversorgungsforschung ein. Damit soll eine prägnante, informative Aufbereitung des Ansatzes erfolgen, die an der Forschung Beteiligte motiviert, partizipative Forschung umzusetzen. Die Darstellung des Stands der Institutionalisierung in Deutschland hat aufgezeigt, dass sich die strukturellen Voraussetzungen in den letzten Jahren verbessert haben, dass aber international in einigen Ländern Entwicklungen stattgefunden haben, die sinnvoll aufgegriffen werden könnten. Als Erfolgsfaktoren einer langfristigen Etablierung des partizipativen Ansatzes wurden die theoretische Fundierung und die empirische Evaluation hervorgehoben. Auch hier bestehen Entwicklungs- und Forschungsbedarfe. Basierend auf dem theoretisch-konzeptionell ausgerichteten ersten Teil des Memorandums wird der folgende zweite Teil in stärkerem Maße auf konkrete Handlungsempfehlungen zur Umsetzung partizipativer Versorgungsforschung im Forschungsalltag eingehen. Dazu wird von typischen Forschungsmethoden berichtet, die in der partizipativen Versorgungsforschung eingesetzt werden und es erfolgt – durch Beispiele veranschaulicht – eine Darstellung der Organisation und konkreten Voraussetzungen von partizipativer Forschung.

## Footnotes

1 Der Einfachheit halber wird im Folgenden „Versorgungsforschung“ als Synonym für „Gesundheitsversorgungsforschung“ verwendet.

2 Mit dem Begriff Patient:innen sind je nach Versorgungssetting auch zukünftige Patient:innen, Rehabilitanden, Bürger:innen, Klient:innen oder Kund:innen gemeint. Patient:innen können Erwachsene oder Kinder/Jugendliche sein.
