# Erratum: DNVF Memorandum Partizipative Versorgungsforschung (Teil 1)

**DOI:** 10.1055/a-2778-7312

**Published:** 2026-02-18

**Authors:** Anna Levke Brütt, Sandra Borgmann, Eva Buchholz, Larissa Burggraf, Jennifer Engler, Florian Fischer, Tim Holetzek, Stefanie Houwaart, Andrea Icks, Franziska Jagoda, Sven Kernebeck, Christine Kersting, Theresia Krieger, Charlotte Kugler, Silke Kuske, Jonas Lander, Melanie Messer, Cathleen Muche-Borowski, Catharina Münte, Anna-Lena Röper, Sandra Salm, Daniel Schindel, Stefanie Schreiter, Sonja Teupen, Sebastian von Peter, Erik Farin-Glattacker

**Affiliations:** 1 Institut und Poliklinik für Medizinische Psychologie, Universitätsklinikum Hamburg-Eppendorf, Hamburg, Germany; 2 Department für Versorgungsforschung, Carl von Ossietzky Universität Oldenburg, Oldenburg, Germany; 3 Institut für Versorgungsforschung und Gesundheitsökonomie, Deutsches Diabetes-Zentrum, Leibnitz Zentrum für Diabetesforschung an der Heinrich-Heine-Universität Düsseldorf, Düsseldorf, Germany; 4 Institut für Versorgungsforschung und Gesundheitsökonomie, Centre for Health and Society, Medizinische Fakultät und Universitätsklinikum Düsseldorf, Heinrich Heine Universität Düsseldorf, Düsseldorf, Germany; 5 Deutsches Zentrum für Diabetesforschung e. V., Neuherberg, Germany; 6 Zentrum für Versorgungsforschung Brandenburg (ZVF-BB), Medizinische Hochschule Brandenburg Theodor Fontane, Brandenburg, Germany; 7 Abteilung Soziologie, Pädagogische Hochschule Schwäbisch Gmünd, Schwäbisch Gmünd, Germany; 8 Institut für Allgemeinmedizin, Goethe-Universität Frankfurt am Main, Frankfurt, Germany; 9 Sachgebiet Kommunikation, Wissenschaft und Gesundheitsförderung, Gesundheitsamt Frankfurt am Main, Frankfurt, Germany; 10 Bayerisches Zentrum Pflege Digital, Hochschule für angewandte Wissenschaften Kempten, Kempten, Germany; 11 Institut für Sozialmedizin und Epidemiologie, Medizinische Hochschule Brandenburg Theodor Fontane, Brandenburg an der Havel, Germany; 12 BRCA-Netzwerk e.V. – Hilfe bei familiären Krebserkrankungen, Bonn, Germany; 13 partieval – Vermittlung partizipativer Kompetenzen, Prozessbegleitung und Evaluation im Bereich Gesundheit GmbH, Aachen, Germany; 14 Institut für Epidemiologie, Sozialmedizin und Gesundheitssystemforschung, Medizinische Hochschule Hannover, Hanover, Germany; 15 Department für Pflegewissenschaft , Universität Witten/Herdecke, Witten, Germany; 16 Fachbereich Gesundheit, Fachhochschule Münster, Münster, Germany; 17 Institut für Allgemeinmedizin und Ambulante Gesundheitsversorgung (iamag), Universität Witten/Herdecke, Witten, Germany; 18 Fachbereich Medizinische Psychologie, Universität zu Köln, Köln, Germany; 19 Institut für Versorgungs- und Gesundheitssystemforschung (IVGF), Medizinische Hochschule Brandenburg Theodor Fontane, Brandenburg, Germany; 20 Forschungsbereich Versorgungs- und Implementierungsforschung, Fliedner Fachhochschule Düsseldorf, Düsseldorf, Germany; 21 Institut für Pflegewissenschaft, Universitätsklinikum Würzburg, Würzburg, Germany; 22 Lehrstuhl für Pflegewissenschaft, Universität Würzburg, Würzburg, Germany; 23 Institut für Allgemeinmedizin, Universitätsklinikum Hamburg Eppendorf, Hamburg, Germany; 24 Institut für Allgemeinmedizin und Palliativmedizin, Medizinische Hochschule Hannover, Hanover, Germany; 25 Deutsche Multiple Sklerose Gesellschaft, Bundesverband e.V., Hannover, Germany; 26 MS Forschungs- und Projektentwicklungs-gGmbH, Deutsches MS-Register, Hannover, Germany; 27 Institut für Medizinische Soziologie, Charité – Universitätsmedizin Berlin, Berlin, Germany; 28 Institut für Soziale Gesundheit , Katholische Hochschule für Sozialwesen Berlin, Berlin, Germany; 29 Klinik für Psychiatrie und Psychotherapie, Charité – Universitätsmedizin Berlin, corporate member of Freie Universität Berlin and Humboldt-Universität zu Berlin, Berlin, Germany; 30 Arbeitsgruppe Methoden in der Versorgungsforschung, Deutsches Zentrum für Neurodegenerative Erkrankungen e.V. (DZNE), Standort Witten, Witten, Germany; 31 Fakultät für Gesundheit, Department für Pflegewissenschaft, Universität Witten/Herdecke, Witten, Germany; 32 Universitätsklinik für Psychiatrie und Psychotherapie Rüdersdorf, Medizinische Hochschule Brandenburg, Rüdersdorf, Germany; 33 Sektion Versorgungsforschung und Rehabilitationsforschung, Universitätsklinikum Freiburg, Medizinische Fakultät, Universität Freiburg, Freiburg, Germany

## Erratum

**DNVF Memorandum Partizipative Versorgungsforschung (Teil 1)**

Anna Levke Brütt, Sandra Borgmann, Eva Buchholz et al. Gesundheitswesen; 10.1055/a-2665-0028

Im oben genannten Artikel wurden mehrere Fehler in den Affiliationen 12, 13, 25 und 26 korrigiert. Die korrekten Affiliationen lauten:

12 BRCA-Netzwerk e.V. – Hilfe bei familiären Krebserkrankungen, Bonn, Germany

13 partieval – Vermittlung partizipativer Kompetenzen, Prozessbegleitung und Evaluation im Bereich Gesundheit GmbH, Aachen, Germany

25 Deutsche Multiple Sklerose Gesellschaft, Bundesverband e.V., Hannover, Germany

26 MS Forschungs- und Projektentwicklungs-gGmbH, Deutsches MS-Register, Hannover, Germany

Die Korrektur wurde in der Online-Version ausgeführt am 18.02.2026.

